# Supplementation with Resveratrol, Piperine and Alpha-Tocopherol Decreases Chronic Inflammation in a Cluster of Older Adults with Metabolic Syndrome

**DOI:** 10.3390/nu12103149

**Published:** 2020-10-15

**Authors:** Raúl Francisco Pastor, Marisa Gabriela Repetto, Fabiana Lairion, Alberto Lazarowski, Amalia Merelli, Zulma Manfredi Carabetti, Isabel Pastor, Elena Pastor, Laura Valeria Iermoli, Carlos Amadeo Bavasso, Roberto Héctor Iermoli

**Affiliations:** 1 Unidad Polifenoles, Vino y Salud, Cuarta Cátedra de Medicina, Hospital de Clínicas “José de San Martín” Facultad de Medicina, Universidad de Buenos Aires, City of Buenos Aires C1120AAF, Argentina; zulmamanfredi@tiempomedico.com.ar (Z.M.C.); ipastor@hospitaldeclinicas.uba.ar (I.P.); epastor@hospitaldeclinicas.uba.ar (E.P.); lauraiermoli@gmail.com (L.V.I.); analisisintegrales@hotmail.com (C.A.B.); riermoli@fmed.uba.ar (R.H.I.); 2 Departamento de Físicoquímica y Química Analítica, Facultad de Farmacia y Bioquímica, Universidad de Buenos Aires; Instituto de Bioquímica y Medicina Molecular (IBIMOL) Consejo Nacional de Ciencia y Tecnología (UBA-CONICET), City of Buenos Aires C1113AAD, Argentina; mrepetto@ffyb.uba.ar (M.G.R.); flairion@ffyb.uba.ar (F.L.); 3 Departamento de Bioquímica Clínica, Facultad de Farmacia y Bioquímica, Universidad de Buenos Aires, Instituto de Fisiopatología y Bioquímica Clínica (INFIBIOC), Buenos Aires C1113AAD, Argentina; nadiatom@ffyb.uba.ar (A.L.); amerelli@ffyb.uba.ar (A.M.)

**Keywords:** metabolic syndrome, chronic inflammation, resveratrol, piperine, alpha-tocopherol

## Abstract

Metabolic Syndrome (MetS) is increasing worldwide regardless of culture, genetic, gender, and geographic differences. While multiple individual risk factors, such as obesity, hypertension, diabetes, and hyperlipidemia, can cause cardiovascular disease (CVD), it is the intercurrence of these risk factors that defines MetS as a cluster that creates an environment for atherosclerosis and other manifestations of CVD. Despite the advances in the knowledge and management of each of the components of MetS, there are two molecular biology processes, chronic inflammation and oxidative stress, which are still underdiagnosed and undertreated. In order to assess the effect of a dietary supplement on chronic inflammation in MetS, we conducted a clinical trial with volunteers receiving a formula composed of resveratrol, piperine and alpha tocopherol (FRAMINTROL^®^), together with their habitual treatment, for three months. The inflammatory state was evaluated by ultrasensitive C reactive protein (US CRP) and ferritin in plasma, and oxygen consumption and chemiluminescence in neutrophils. The results showed that ferritin decreased by 10% (*p* < 0.05), US-CRP by 33% (*p* < 0.0001), oxygen consumption by 55% (*p* < 0.0001), and spontaneous chemiluminiscence was by 25% (*p* < 0.005) after treatment. As far as we know, this is the first study showing a chronic inflammation decrease in MetS patients due to the administration of a biopower Resveratrol-piperine and alpha tocopherol dietary supplement together with conventional therapy.

## 1. Introduction

According to the World Health Organization, obesity, a common denominator of MetS, has in general increased three-fold worldwide since 1975. Lifestyle changes, such as a marked reduction in physical activity levels and an increased consumption of high-calorie foods, have been conducive to the increased prevalence of obesity.

Epidemiological data from 2016 show that 39% of individuals over 18 were overweight and 13% were obese. It has been shown that overweight and obesity are risk factors for the noncommunicable chronic diseases (NCCDs) included in MetS. Consequently, the deterioration in quality of life for both patients and families added to the high medical and social costs are a great concern for health care systems.

MetS is a clinical disorder that is defined by the co-occurrence of other CVD risk factors, such as central obesity, abnormalities of glucose metabolism or diabetes, dyslipidemia and arterial hypertension. No standardized global data are available on the prevalence of MetS because diagnostic criteria vary in different guidelines [1]. In Argentina, a systematic review carried out with urban populations, with a mean age of 46.2, found a prevalence of MetS of 27.5% for both genders, higher in males than females, at 29.4% vs. 27.4% respectively [2].

In another systematic review conducted in Latin American countries, the prevalence reached 24.9% and was slightly higher in females (25.3%) than in males (23.2%); individuals over the age of 50 had the highest prevalence [3].

A meta-analysis conducted in China found that the general prevalence of MetS in individuals over 15 years of age was 24.5%, of which 19.2% were males and 27% were females. This prevalence increased with aging, being at around 32.4% in individuals over 60 [4].

A systematic review conducted in Brazil found an average prevalence of 29.6%, and the indigenous and rural populations were the most and the least affected with 65.3% and 14.9%, respectively [5]. In the USA, based on data from the National Health and Nutrition Examination Survey (NHANES) between 2003–2012, the prevalence of MetS was at 33%, higher in females than in males, at 35.6% vs. 30.3% respectively [6].

A systematic review conducted in Middle East countries found an average prevalence of MetS of 25% [7]. The PROMETS trial in Portugal found a prevalence of 36.5%, and MetS was slightly more common in females than in males, at 40.7% vs. 39% respectively [8].

It has been well proven that obesity features a chronic low grade inflammatory state and that the cumulative molecular damage caused by the oxidative metabolism plays a key role in the pathogenesis of age-related conditions, also contributing to increased inflammation and oxidative stress levels [9,10]. These two processes are interdependent and often share signaling pathways and biochemical processes that accelerate aging from the onset of chronic diseases included in MetS. Redox homeostasis is maintained in physiological situations at the expense of the antioxidant regulation of the concentrations of oxidant species, and in terms of molecular biology it involves intracellular protein signaling and transcription mechanisms [11]. When these mechanisms are altered or absent, an excessive accumulation of biomarkers of inflammation and oxidative stress occurs, with byproducts of biomolecular oxidation, and signaling pathways are therefore altered [11,12].

Based on these findings, several clinical trials have investigated whether vitamin antioxidants might prevent NCCDs [13,14]. However, the obtained results were generally disappointing because, although some of the studies found benefits for health, others did not obtain any results or even found deleterious effects [15]; this is because, in some cases, biology uses free radicals as signaling pathways, and massive elimination might partly account for this failure, leading to what is known as the antioxidant paradox of vitamins [16,17,18].

The MetS is one of the maximum expressions of chronic inflammation, as several chronic inflammatory conditions coexist. This inflammatory syndrome, which accelerates aging, is still generally underdiagnosed and undertreated in medical practice. For this reason, the aim of our research was to assess the effect of a Resveratrol + Piperine + Alpha-tocopherol (FRAMINTROL^®^) dietary supplement on chronic inflammation in MetS.

## 2. Materials and Methods

Between May and December 2019, we publicly invited out-patients with MetS to participate in a clinical trial approved by the Ethics Committee of the University of Buenos Aires, Clinics Hospital. These included twenty-two patients (13 males and nine females), mean age 68 ± 4.7 years, diagnosed with MetS according to the global harmonized definition of having three of the five following characteristics: [19] 1. Blood glucose levels over 100 mg/dL or drug therapy for high blood glucose levels; 2. HDL Cholesterol < 40 mg/dL in males or < 50 mg/dL in women, or drug therapy for low HDL cholesterol levels; 3. Blood triglycerides > 150 mg/dL or drug therapy for high triglyceride levels; 4. Waist circumference > 102 cm for males or > 88 cm for females; and 5. Blood pressure > 130/85 mmHg or antihypertensive drug therapy.

At the first interview, the participants of this clinical trial #26122018 approved by the Ethics Committee of the “José de San Martín” Clinics Hospital University of Buenos Aires were presented, in accordance with the 1964 Helsinki Declaration and the later updated versions of Tokyo (1975), Venetia (1983), Hong Kong (1989), Somerset West (1996), Edinburgh (2000), Washington (2002), Tokyo (2004), Korea (2008) and Brazil (2013), and also according to the personal data protection Act N° 25.326; then, the inclusion criteria were analyzed, and informed consent was obtained.

The Ethics Committee, through the approved protocol, established as a condition that the background treatments for NCCDs, oral hypoglycemic agents, antihypertensives or medical therapy for dyslipidemia should not be suspended and should remain unchanged during the trial. The average baseline data of all the patients are included in Table 1.

As the participation of the five different CVD risk factors within MetS varies among patients, we presented, as shown in Table 2, the percentage of each condition in MetS. Hypertension was the most prevalent entity.

Table 3, Table 4 and Table 5 show the clinical data of each patient at the beginning of the trial, where the different MetS expressions can be observed. As can be seen, some risk factors were controlled by treatment and others were not.

Patients started treatment after a two-week washout of dietary supplements (antioxidants, vitamins or minerals), control visits were scheduled for 30, 60 and 90 days, and the treatment included two pills per day of a biopower formula of resveratrol 50 mg + piperine 5 mg + alpha tocopherol 25 mg (FRAMINTROL^®^), one with each main meal.

According to the design of the study, each patient was their own matched control, in order to prevent interindividual variables and the bioavailability of the active ingredient. Blood and urine tests were performed twice, at the beginning and at the end of the treatment. The following parameters in plasma were assessed: glycemia, HDL Cholesterol, triglycerides, ferritin, US CRP, and the oxygen consumption and spontaneous chemiluminescence activity in isolated neutrophils.

Biochemical measurements were obtained using a diagnostic kit from Roche Laboratories, measured with Hitach Covas C311. Hematological measurements were made using LABIX reactive kits measured with SEAC HECO.

### 2.1. Samples and PMN Isolation

The venous blood samples were obtained with heparin. PMNs were isolated from blood samples in the Hematology Laboratory of the Clinical Biochemistry Department of “José de San Martín” Clinics Hospital using Ficoll–Hypaque method and exposed to hypotonic shock with a sterile NaCl solution (0.2% w/v) and the same volume of sterile NaCl solution (1.6% w/v). This suspension of PMNs was centrifuged for 10 min at 450 g at 20 °C, the supernatant fluid was discarded, and the pellet was resuspended in 10 mL of sterile RPMI 1640 medium at 37 °C [20].

### 2.2. Neutrophils Viability

PMNs’ viability was tested with a vital staining Trypan Blue solution (0.4%), and only cell suspensions with a viability >95% were used.

### 2.3. PMNs Count and Viability

Total and differential cell accounts of viable PMNs in suspension, were developed using the Sysmex XP-300™ Automated Hematology Analyzer (Sysmex^®®^ XN-Series, Chuo-ku, Kobe, Japan) Sysmex Corporation, Chuo-ku, Kobe, Japan, in a mode to distinguish the mononuclear (MN) cells from neutrophils. These counts were confirmed manually with Neubauer’s chamber, using a 1/400 dilution of the PMNs suspension in Turk’s solution (1–2% acetic acid with aqueous methylene blue). PMNs’ viability was measured with an equal volume of Trypan Blue solution (T8154-Sigma-Aldrich) using a 1/100 dilution of the abovementioned neutrophil suspension. The mononuclear cells/neutrophils ratio as well as cell viability was determined before and after each experiment. PMN samples were sent to the School of Pharmacy and Biochemistry for oxygen consumption and chemiluminescence.

### 2.4. PMN Oxygen Consumption

PMN oxygen uptake was determined polarographically by high resolution respirometry, using a Clark-type electrode (Hasantech Oxygraph System DW1, Norfolk, England) Hasantech Instruments Ltd, Norfolk, England, thermostated at 37 °C, with human PMN (10^6^/mL) in PBS supplemented with 0.9 mM CaCl_2_, 0.5 mM MgCl_2_ and 7.5 mM glucose (PBSG). For the assay, respiratory buffer (for a 1 mL final volume) was placed in the electrode chamber, and the rate of oxygen consumption was calculated from the initial time course and expressed as nmol of oxygen/min/10^6^ cells [21].

### 2.5. PMN Chemiluminescence

Spontaneous chemiluminescence in fresh PMNs was measured with a photon counter developed by Chance, Sies and Boveris (1979) in the Johnson Research Foundation of the University of Pennsylvania (Philadelphia, PA, USA). The results were expressed in cps/mL PMN (cps: counts per second, 1 cps corresponds to about 10^3^ photons per second) [22,23,24].

### 2.6. Statistics

The statistical analysis was performed using the Student *t*-test for paired samples (Prim 7.0, GraphPad, San Diego, CA, USA). The Student *t*-test was selected to evaluate a single biomarker at a time in eight series of paired data (D0 vs. D90 for plasma ferritin and US CRP, in neutrophils, oxygen consumption and spontaneous chemiluminescence) for each patient before and after the intervention.

## 3. Results and Discussion

The 22 patients with MetS showed a good tolerance to the treatment with two pills a day of the Resveratrol 50 mg + piperine 5 mg + alpha-tocopherol 25 mg (FRAMINTROL^®^) formula, with no side effects.

**Figure 1:**
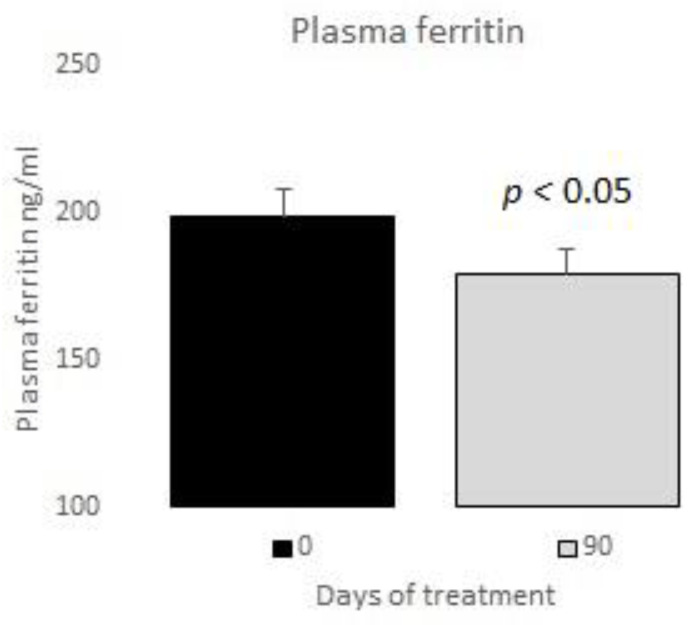
Plasma ferritin basal vs. 90 days of treatment with resveratrol, piperine and alpha-tocopherol in MetS patients.

**Figure 2:**
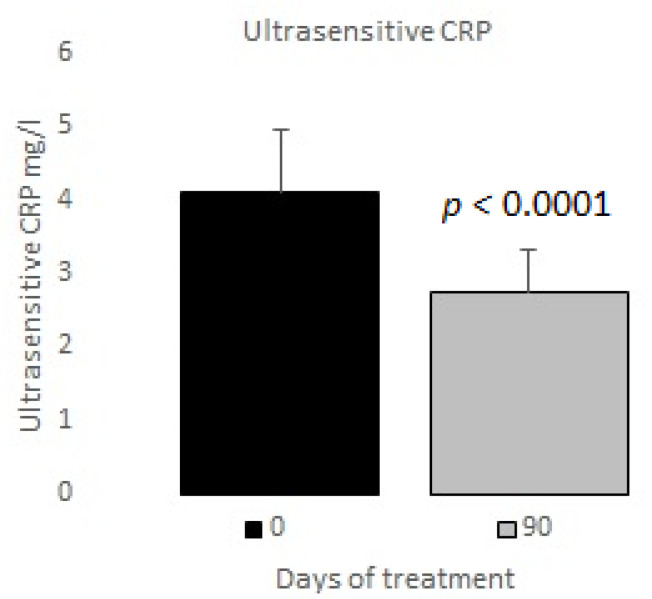
Ultrasensitive C reactive protein basal vs. 90 days of treatment with resveratrol, piperine and alpha-tocopherol in MetS patients.

**Figure 3:**
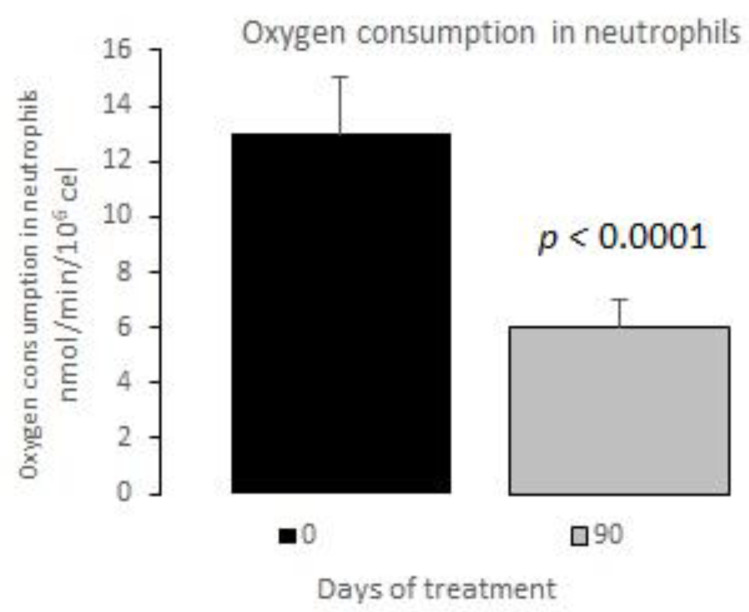
Oxygen consumption in neutrophils basal vs. 90 days of treatment with resveratrol, piperine and alpha-tocopherol in MetS patients.

**Figure 4:**
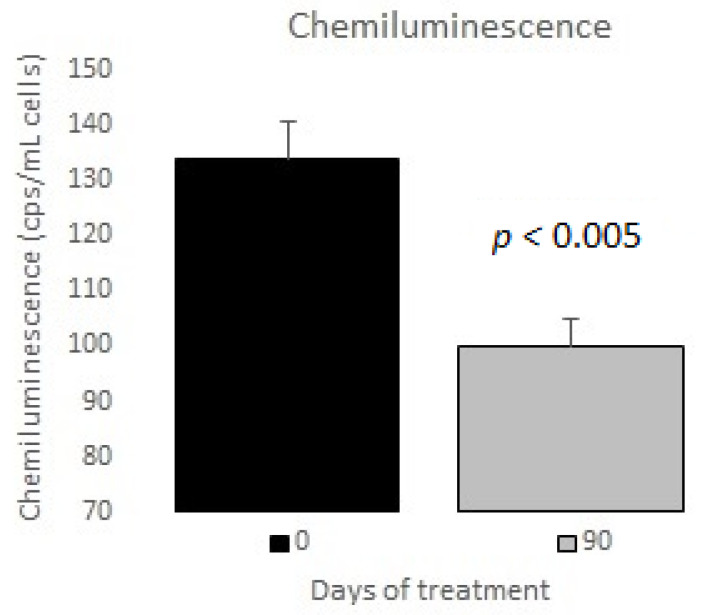
Chemiluminescence in neutrophils basal vs. 90 days of treatment with resveratrol, piperine and alpha-tocopherol in MetS patients.

The measurements of the inflammatory state appear in Table 6. The observed baseline and post-treatment variations were all beneficial, plasma ferritin levels decreased significantly by 10%, and US CRP decreased in plasma by 33%, evidencing a highly significant drop (Figure 1 and Figure 2); in neutrophils, oxygen consumption decreased by 55% and spontaneous chemiluminescence by 25% after 90 days of treatment; both decreases were highly significant (Figure 3 and Figure 4).

Arterial hypertension is one of the most prevalent chronic cardiovascular conditions worldwide, and it was also the most prevalent in our MetS study population (91% of our patients) (Table 2). It has been proven that low-grade chronic inflammation in arterial hypertension is present since the onset of the disease.

Although the etiology of essential arterial hypertension is still unknown, it has been found that both the innate and adaptive immune systems participate interdependently in the development of sustained high blood pressure, as well as in endothelial, kidney and target organ damage [25].

The phenomenon that triggers arterial hypertension has not been elucidated; however, it is known that pro-hypertensive stimuli such as Angiotensin II or excessive use of salt make lymphocytes T proinflammatory, which increases the release of inflammatory cytokines as well as oxidative stress levels [26,27].

Increased metabolic activity in neutrophils in patients with MetS, as measured indirectly in oxygen consumption, might signal the intense activation of these inflammatory cells.

Obesity and diabetes were reported in 86% and 73% of the patients with MetS, respectively (Table 2). The physical effects related to the increase in adipose tissue caused by obesity provide many intrinsic signals produced by mechanical stress, hypoxia and adipocyte death that are able to initiate the inflammatory response. It has been shown that, as adipose tissue increases during obesity, inflammation and macrophage-innate immune cell-accumulation increase as well and that this infiltration reaches as much as 40% of all the adipose tissue cells [28,29]. 

The mechanical stress that triggers the inflammation in adipose tissue in obese individuals was first published in the early 1990s. This investigations showed that the adipose tissue of obese volunteers presented inflammatory changes with an increased release of cytokines mediated by tumor necrosis factor-alpha (TNF-α), and this finding correlated with the inhibition of the insulin receptor substrate and consequently with an increased insulin resistance [30]. This finding has also been proven in basic research through reverse engineering, where TNF-α block increases sensitivity to insulin and improves glucose metabolism [30]. These findings confirm the significant activation of inflammatory processes by the innate and adaptive immune systems [31] in obesity, which constitute risk factors for increased insulin resistance and diabetes. In brief, lipid accumulation in adipose tissue in obese patients triggers an inflammatory response, resulting in an increased release of several cytokines [32,33]. It has also been evidenced that dyslipidemia induces an inflammatory response due to the activation of the immune system. Persistently high oxidized LDL cholesterol levels in plasma drive the production of interleukin 1 and 6 and therefore drive increases in US CRP levels [34]. High plasma ferritin is a biomarker of MetS. It was found that plasma ferritin values were correlated directly with different clinical expressions of MetS with three, four or five CVD risk factors [35,36]. In other words, there were more CVD risk factors with more plasma ferritin. Furthermore, it has been proven that, of all the MetS components, triglycerides and hyperglycemia are the variables that correlate the most with high plasma ferritin levels [37]. C reactive protein (CRP) is a protein of the pentraxin family, synthesized and released by endothelial cells in atherosclerotic plaques, hepatocytes, lymphocytes and macrophages, mainly in response to the increase of interleukin 7 (IL 7). It has been evidenced that US CRP in plasma is a biomarker of the inflammatory system in downstream MetS, and potentially a good biomarker for monitoring treatment results [12,38,39].

In our study, the post-treatment decreases in plasma ferritin and US CRP reached 10% and 33%, respectively (Figure 1 and Figure 2). Thus, we believe that those variables are a potential tool for the clinical control of patients’ outcomes.

Piperine is a bioactive alkaloid that gives black pepper its pungency. It has a broad spectrum of action in molecular biology, as an antioxidant, anti-inflammatory, antiangiogenic, antibacterial and immunomodulatory agent. Piperine may be naturally extracted from black pepper, with a 6% to 13% yield. Despite its many healthy properties, the use of piperine in human health is still limited due to its scarce availability and low solubility in water [40,41]. However, piperine has been shown to be a resveratrol booster, since the oral administration of a formula of piperine plus resveratrol (10/100 mg/kg, respectively) increases the bioavailability of resveratrol by 1544% when compared to resveratrol alone [42].

Alpha-tocopherol has been shown to have anti-inflammatory effects due to the decreased production of anti-inflammatory cytokines IL-1 beta IL-6 and chemokine IL-8, together with the neutralization of alpha-TNF [43]. In obese individuals with and without diabetes, MetS is related to chronic systemic inflammation and low levels of alpha-tocopherol [44].

Neutrophils are the main intracellular source of superoxide anion (O_2_^−^) and hydrogen peroxide (H_2_O_2_) in the inflammatory processes associated with human obesity and metabolic syndrome. These reactive oxygen species are mainly generated in PMN cells through NADPH oxidase complex activity. The rate of oxygen consumption measured in the Clark-type electrode indicates the electron transfer process for generating O_2_^−^ and, therefore, H_2_O_2_ production. Spontaneous chemiluminescence is a useful approach for determining the occurrence of oxidative stress in either cells or tissues. Spontaneous chemiluminescence determination is a noninvasive, nondestructive indirect assay based on measuring the light emission from an excited state to a basal state of singlet oxygen (^1^O_2_) and peroxyl radicals (ROO), reactive and toxic products of phospholipid peroxidation. The number of emitted photons depends on the steady-state concentration of ^1^O_2_, which is an indicator of the oxidative damage due to the final stage of the phospholipid oxidation in the chain reaction [22,23,24]. Resveratrol supplementation decreased the oxygen consumption and chemiluminescence levels in PMN of patients with MetS, indicating an anti-inflammatory and antioxidant effect after treatment (Figure 3 and Figure 4).

## 4. Conclusions

Metabolic syndrome is a clinical entity with an increasing incidence and high prevalence worldwide, including several CVD risk factors; obesity, arterial hypertension, hyperglycemia and dyslipidemia have a common denominator, i.e., the chronic activation of the immune system, with inflammatory processes which damage the molecular biology and accelerate aging. In our research, we used a biopower formula containing three active ingredients: Resveratrol + Piperine + Alpha-Tocopherol (FRAMINTROL^®^) in the management of patients with MetS.

The results of this treatment evidenced (1) a significant decrease in plasma ferritin levels and a highly significant decrease in US-CRP levels; (2) Plasma ferritin and US CRP might be good biomarkers of inflammation for the clinical follow up of patients with MetS; (3) A highly significant decrease in the oxygen consumption and spontaneous chemiluminescence of polymorphonuclear cells might be indicative of a remarkable drop in the proinflammatory metabolism of these cells of the immune system and of decreased levels of oxidant reactive species; (4) Reducing chronic inflammation in MetS patients should be a new prevention goal to decrease CVD risk factors.

## Figures and Tables

**Table 1:** Patient average baseline data.

Patients (Male–Female)	22 (M 13–F 9)
Age (years)	68 ± 4.7
Weight (kg)	82 ± 17.5
Body Mass Index (kg/m^2^)	29.25 ± 3.4
Systolic blood pressure (mmHg)	135 ± 25.85
Diastolic blood pressure (mmHg)	86 ± 18.32
Waist circumference (cm)	109 ± 23.30
Blood glucose (mg/dL)	103 ± 21
HDL Cholesterol (mg/dL)	57.95 ± 12.32
Triglycerides (mg/dL)	126 ± 26.86
Plasma ferritin (ng/mL)	198.45 ± 38.11
Ultrasensitive C reactive protein (mg/L)	4.10 ± 0.87
Oxygen consumption (nmol O_2_/min/ 10^6^ cells)	13 ± 2
Chemiluminescence (cps/mL cells)	134 ± 47

**Table 2:** Relative participation and percentage of the five CVD risk factors in the MetS patients.

	Arterial Hypertension	Waist Circumf	Triglycerides	HDL Cholesterol	High Glucose or Diabetes	Patients/CVD Risk Factors in MetS
Patients	20/22 91%	19/22 86%	18/22 82%	18/22 82%	16/22 73%	10/5 5/4 7/3

**Table 3:** Baseline data of seven MetS patients with 3/5 CVD risk factors. The shadowed cells indicate the treated conditions. BP, Blood Pressure: (mmHg); WC, waist circumference (cm); TG: Triglyderides (mg/dL); HDL Chol., HDL cholesterol (mg/dL); BG, Blood Glucose (mg/dL).

Patient ID	BP	WC	TG	HDL Col.	BG
HB	180/100	114	71	48	97
CF	120/80	88	105	77	99
MG	148/86	93	64	51	91
PH	105/78	107	90	81	106
HL	116/60	98.5	169	45	90
RS	130/90	110	90	61	90
ES	130/96	101	133	40	98

**Table 4:** Baseline data from five MetS patients with 4/5 CVD risk factors. The shadowed cells indicate the conditions under treatment.

Patient ID	BP	WC	TG	HDL Chol.	BG
BH	150/90	114	79	39	105
AD	160/100	99	57	77	101
LV	120/80	104	107	76	90
EV	130/80	123	282	39	89
MG	100/86	93	64	51	91

**Table 5:** Baseline data from 10 MetS patients with 5/5 CVD risk factors. All of them were receiving treatment for their baseline conditions.

Patient ID	BP	WC	TG	HDL Chol.	BG
JB	140/90	112	65	60	105
RB	140/92	140	131	37	136
SN	120/70	99	50	66	99
CL	160/84	128	155	79	116
JP	138/86	128	238	64	104
AS	120/80	114.5	354	30	111
MS	130/90	119	100	68	119
ET	130/90	110	202	43	110
LA	140/98	95	104	89	110
HA	170/100	103	58	54	102

**Table 6:** Variables of the inflammatory state; in plasma: ferritin and US CRP, and in neutrophils: baseline and post-treatment (after 90 days of resveratrol, piperine y alpha-tocopherol) oxygen consumption and chemiluminescence.

Biomarkers/ Basal vs. Final	Basal	Final	*p*	Δ %
Plasma ferritin (ng/mL)	198.45 ± 38.11	178.75 ± 21.90	<0.05	10
Oxygen consumption (nmol O_2_/min/ 10^6^ cells)	13 ± 2	6 ± 1	<0.0001	55
Ultrasensitive C reactive protein (mg/L)	4.10 ± 0.87	2.74 ± 0.59	<0.0001	33
Chemiluminescence (cps/mL cells)	134 ± 47	100 ± 22	<0.005	25
